# How often do primary care veterinarians record the overweight status of dogs?*

**DOI:** 10.1017/jns.2014.42

**Published:** 2014-12-08

**Authors:** Nicola C. Rolph, Peter-John M. Noble, Alexander J. German

**Affiliations:** 1 School of Veterinary Science, University of Liverpool, Leahurst Campus, Chester High Road, Neston, Wirral CH64 7TE, UK; 2 Department of Obesity and Endocrinology, University of Liverpool, Leahurst Campus, Chester High Road, Neston, Wirral CH64 7TE, UK

**Keywords:** Obesity, Canine nutrition, Practice surveillance, BCS, body condition score, NSAID, non-steroidal anti-inflammatory drug, OW, overweight

## Abstract

Obesity is a prevalent medical condition in dogs caused by the excess accumulation of fat, with negative effects on quality of life, longevity and the risk of developing associated pathologies. However, it is unclear how frequently first-opinion veterinarians record dogs as overweight (OW) or obese in medical records, and what factors determine when they do. Data sourced through the Small Animal Surveillance Network were used to determine the relative frequency of recording OW status (obesity or OW) in dogs presented to the UK first-opinion practices. Cases were identified using a search of clinical record-free text for relevant keywords. A case–control study was then conducted, comparing dogs where the OW status was recorded with a control group of obese dogs with no diagnosis recorded. Of 49 488 consultations, the OW status was recorded in 671 dogs (relative frequency 1·4 %). Using multiple logistic regression, the OW status of a dog was more likely to be recorded when the consultation was for osteoarthritis (OR 5·42; 95 % CI 2·09, 14·07; *P* < 0·001) or lameness (OR 2·02; 95 % CI 1·20, 3·42; *P* = 0·006). Furthermore, the OW status was more commonly recorded in dogs that were members of a practice health scheme (OR 5·35; 95 % CI 1·57, 18·17; *P* = 0·04) and less commonly recorded in microchipped dogs (OR 0·43; 95 % CI 0·41, 0·91; *P* = 0·02). These results suggest that OW and obesity are underdiagnosed in the first-opinion practice. However, a presentation for orthopaedic disease appears a key prompt for recording the OW status. Further studies are now warranted to determine the reasons for such marked underdiagnosis.

Obesity is defined as a disease in which excess body fat has accumulated such that health may be adversely affected^(^^1^^)^, and is one of the most common medical diseases in dogs^(^^2^^,^^3^^).^ Recent UK studies put the prevalence of overweight (OW) and obese dogs between 52^(^^3^^)^ and 59 %^(^^4^^)^, with a rising trend and worldwide problem suggested^(^^2^^,^^4^^)^.

Owners commonly misperceive the body shape of dogs, and this is the most marked for OW dogs^(^^5^^–^^7^^)^. Thus, it is vital for veterinarians to provide guidance as to what constitutes ideal body condition, and support this with effective education and communication regarding obesity prevention^(^^8^^)^. Despite this, a previous study demonstrated that veterinarians rarely perform body condition scores (BCS) on dogs in primary care practice, suggesting that they rarely discuss the issue of OW status with owners. There are a number of possible reasons for this, including more pressing medical problems^(^^9^^)^, the fact that the owner is obese^(^^10^^)^ or time limitations^(^^11^^)^.

The main aim of the present study was to determine the frequency of veterinarians recording dogs as obese or OW in the first-opinion practice. The study used a larger, more representative population of dogs than previous studies, and utilised data obtained from veterinary practice management software. The aim of a further study was to identify factors that were associated with the likelihood of OW status being recorded.

## Experimental methods

This was a retrospective, correlational case–control study. Data were collected from practices using a compatible version of practice management software (Premvet v03.02.12) following a positive response to a postal request. seventy-four practices were approached, recruiting 16/59, 3/7, 0/6 and 0/2 practices in England, Wales, Scotland and Northern Ireland, respectively (total nineteen practices comprising forty-two premises). Data were collected between 10 May 2010 and 8 August 2011. Data were stored in, and retrieved from, a relational database (MySQL, Oracle). In total, 73 000 small animal consultations were contained within the database, of which 49 488 related to dogs.

All client-identifying data (e.g. owner name, address, postcodes and practice details) were either removed or coded to maintain anonymity of participants. Information was then exported to a spreadsheet application (Microsoft Excel version 10.6871.6870, Microsoft Corporation) and included signalment data such as breed, date of birth, sex, microchipping and insurance status, as well as free text written by the veterinarian about the consultations and the date conducted. Consultation duplicates were identified and removed prior to data analysis.

Cases (e.g. consultations where the dog was identified as having an OW status (i.e. the text included a term that suggested the dog was either obese or OW)) were selected from the total 49 488 canine consultations by a search of the clinical record free text using words or phrases that correspond to likely diagnoses by veterinarians (Supplementary data). The number of initial case consultations identified was used to calculate the relative frequency of recording the OW status in dogs within the Small Animal Surveillance Network database. Consultations with missing information on date of birth, consultation date, breed, sex and neuter status were then excluded, since these variables were required for matching purposes. Furthermore, consultations with mixed-breeds and animals less than 2 years of age were excluded, since variance in weight within these categories might have affected valid selection of control animals. The free text for each consultation was then assessed, in order to confirm all details, and 231 consultations remained.

A matched group of controls (e.g. consultations where the dog was likely to be OW or obese, but this was not stated in the free text (i.e. none of the search terms were present)) was also identified). Given that BCS was recorded in <25 % of dogs, it was not possible to use this measure to identify OW or obese control dogs. Instead, OW control dogs were identified using body weight data: for each case, all similar dogs (e.g. with the same breed, age, sex and neuter status) were first identified in the database, and those with a body weight in the upper quartile of weight within this group were then selected (calculated by a formula in Microsoft Excel). For each case, three control dogs were selected from this group using the random series generator of a statistical software package (StatsDirect, version 2.6.8, StatsDirect Ltd.). If it was not possible to match a case to three controls, the case was removed. This method ensured that control dogs were matched to cases for breed and sex, and were among the heaviest dogs with the same signalment. Ultimately, there were 146 remaining cases and 438 matched controls. Signalment factors (age, sex, neuter status, breed (i.e. all breeds with more than ten dogs in the case group)) and body weight were compared between groups to confirm adequacy of group matching.

For all cases and controls, additional variables recorded were: vaccination appointment; parisiticide prescribed (including both endo- and ecto-parasiticides); non-steroidal anti-inflammatory drug (NSAID) prescribed; People's Dispensary for Sick Animals (a charity subsidising veterinary care for people receiving housing benefit or local government tax) registered; pet health club member; osteoarthritis present; lameness present; vomiting noted; diarrhoea noted; anal sacs expressed; aggressive behaviour noted; and abdominal palpation noted. Consultations were already defined in the database as ‘yes’ or ‘no’ for the further variables of microchipping and whether or not the animal was insured. The absolute frequencies of each variable for both cases and controls were recorded, and their relative frequencies calculated. Data were then exported to a statistical software package (StatsDirect, version 2.6.8) and binary logistic regression was used to calculate OR and *P* values for each variable. Initially, simple regression was conducted. Thereafter, a multiple regression model was constructed, which initially included all variables that were *P* ≤ 0·20. The model was subsequently refined by backwards-stepwise elimination of the least significant variable at each round. Variables were retained in the final model, either if they were significant in their own right, or if removal lead to a significant (>10 %) change in the effect of the other variables. The level of statistical significance was set at *P* < 0·05, for two-sided analyses.

## Results

### Relative frequency of veterinarians recording overweight status

Based upon the search terms, the OW status of a dog was recorded in 671 of 49 488 consultations, a relative frequency of 1·4 %.

### Case and control group summaries

In the case group, median age was 7 years (range 2–15 years), and this was similar to the age of controls (median 7 years, range 2–16 years). In both groups, 49 % were male (cases 72/146; controls 216/438) and 51 % were female (cases 74/146, controls 222/438), while 75 % were neutered in both groups (cases 110/146, controls 330/438). In cases, median weight was 25·3 kg (range 4·1–57·0 kg), while the median weight of control dogs was 24·9 kg (range 4·1–54·4 kg). A total of twenty-two breeds were included, with Labrador Retrievers (thirty-three, 23 %), Jack Russell Terriers (twenty-one, 14 %), Staffordshire Bull Terriers (fifteen, 10 %), border collies (eleven, 8 %) and West Highland white terriers (ten, 7 %) contributing the most consultations.

### Risk factors for recording overweight status

With simple logistic regression analysis (Table 1), factors associated with a veterinarian recording of OW status included discussing osteoarthritis, discussing lameness, being microchipped and being a member of a practice health scheme. The same factors remained significant in the final multiple regression model. No other factors examined were of significance, either with simple or multiple regression.

**Table 1.** Simple and multiple logistical regression analysis on factors associated with a veterinarian recording overweight status

		Cases (146)	Controls (438)	OR (95 % CI)	*P* value
Simple regression					
Age (years)*		7 years (2–16 years)	7 years (2–12 years)	0·99 (0·94, 1·05)	0·799
Sex	Female	74 (51 %)	222 (51 %)	(Reference level)	–
	Male	72 (49 %)	216 (49 %)	1·00 (0·69, 1·45)	>0·999
Neuter status	Entire	36 (25 %)	108 (25 %)	(Reference level)	–
	Neutered	110 (75 %)	438 (75 %)	1·00 (0·65, 1·54)	>0·999
Labrador	No	113 (77 %)	99 (77 %)	(Reference level)	–
	Yes	33 (23 %)	339 (23 %)	1·00 (0·64, 1·56)	0·998
JRT	No	125 (86 %)	375 (86 %)	(Reference level)	–
	Yes	21 (14 %)	63 (14 %)	7·37 (0·00, ∞)	>0·999
SBT	No	131 (90 %)	393 (90 %)	(Reference level)	–
	Yes	15 (10 %)	45 (10 %)	1·00 (0·54, 1·85)	>0·999
Border collie	No	135 (92 %)	405 (92 %)	(Reference level)	–
	Yes	11 (8 %)	33 (8 %)	1·00 (0·49, 2·03)	>0·999
WHWT	No	136 (93 %)	408 (93 %)	(Reference level)	–
	Yes	10 (7 %)	30 (7 %)	1·00 (0·48, 2·10)	>0·999
Vaccinated	No	135 (92 %)	409 (93 %)	(Reference level)	–
	Yes	11 (8 %)	29 (7 %)	1·15 (0·56, 2·37)	0·705
Insured	No	103 (71 %)	275 (63 %)	(Reference level)	–
	Yes	43 (29 %)	163 (37 %)	0·70 (0·47, 1·06)	0·090
Microchipped	No	87 (60 %)	212 (48 %)	(Reference level)	–
	Yes	59 (40 %)	226 (52 %)	0·64 (0·44, 0·93)	0·020
Practice health scheme	No	140 (98 %)	433 (96 %)	(Reference level)	–
	Yes	6 (2 %)	5 (4 %)	3·71 (1·12, 12·35)	0·032
PDSA registered	No	141 (97 %)	409 (93 %)	(Reference level)	–
	Yes	5 (3 %)	29 (7 %)	0·50 (0·19, 1·32)	0·161
Osteoarthritis discussed	No	133 (91 %)	431 (98 %)	(Reference level)	–
	Yes	13 (9 %)	7 (2 %)	6·02 (2·35, 15·39)	<0·001
Lameness consultation	No	117 (80 %)	391 (89 %)	(Reference level)	–
	Yes	29 (20 %)	47 (11 %)	2·06 (1·24, 3·42)	0·005
Vomiting consultation	No	138 (95 %)	402 (92 %)	(Reference level)	–
	Yes	8 (5 %)	36 (8 %)	0·65 (0·29, 1·43)	0·281
Diarrhoea consultation	No	143 (98 %)	414 (95 %)	(Reference level)	–
	Yes	3 (2 %)	24 (5 %)	0·36 (0·11, 1·22)	0·101
Anal sacs expressed	No	139 (95 %)	403 (92%)	(Reference level)	–
	Yes	7 (5 %)	35 (8 %)	0·58 (0·25, 1·34)	0·200
Parasiticide prescribed†	No	129 (88 %)	390 (89 %)	(Reference level)	–
	Yes	17 (12 %)	48 (11 %)	1·07 (0·59, 1·93)	0·820
NSAID prescribed	No	45 (69 %)	115 (74 %)	(Reference level)	–
	Yes	101 (31 %)	323 (26 %)	1·25 (0·83, 1·89)	0·284
Multiple regression					
Practice health scheme				5·35 (1·57, 18·17)	0·007
Microchipped				0·43 (0·41, 0·91)	0·015
Osteoarthritis discussed				5·42 (2·09, 14·07)	<0·001
Lameness consultation				2·02 (1·20, 3·42)	0·008

Frequencies and relative frequencies by case and control groups of dogs either positive or negative for each variable are displayed in the tabular format, with corresponding OR, 95 % CI and *P* values. JRT, Jack Russell terrier; SBT, Staffordshire bull terrier; WHWT, West Highland white terrier; PDSA, People's Dispensary for Sick Animals; NSAID, non-steroidal anti-inflammatory drug.

*Age data expressed as median (range).

†Parasiticide medication prescribed during the consultation, and included endoparasitic and ectoparasitic drugs.

## Discussion

The present study has demonstrated that veterinarians in the first-opinion practice rarely record the OW status of dogs. In only 1·4 % of consultations, did the contemporaneous free-text entry indicate that dogs were either OW or obese. These data are consistent with those of a previous study, which demonstrated that veterinarians rarely perform weight measurements and body condition scoring^(^^12^^)^. However, the present study expands upon the previous work because it was much larger (e.g. 49 488 *v.* 148 dogs), the study population more representative, data were taken directly from the veterinary practice management software, and it asked a different research question: the previous study asked how frequently body weight is measured and a BCS is performed in primary care practice; in contrast, the present study asked how frequently veterinarians record the OW status of a dog with their owner, and what prompts them to do this. Both studies also used different research methodology. In the previous study, the computer records sent to a second-opinion veterinary hospital were reviewed for evidence of body weight measurements and BCS. In the present study, data were obtained as part of a national surveillance project, and directly collected from the practice management software. This enabled patient records to be examined for evidence of terms associated with the OW status. Despite these differences, both studies confirm that veterinarians infrequently assess the OW status in dogs that they examine.

Given that BCS was uncommonly recorded, it was not possible to determine the prevalence of OW status in the present population. However, if prevalence is similar to the expected prevalence in UK dogs, based on the recent studies^(^^3^^,^^4^^)^, this suggests that the condition is markedly underreported. All such findings have wide-reaching consequences for the veterinary profession, since they suggest that many dogs are not treated despite the well-recognised health consequences, which include increased disease risk^(^^13^^)^, shortened lifespan^(^^14^^)^, metabolic dysfunction^(^^15^^)^ and decreased quality of life^(^^16^^)^. Interestingly, veterinarians have specified obesity as one of the main issues they could do more for as a profession^(^^17^^)^, indicating possible awareness of underdiagnosis. Finding the most appropriate means of communicating with owners regarding the topic of obesity^(^^18^^,^^19^^)^ might be a useful strategy in ensuring more veterinarians are prepared to actively engage owners with OW dogs.

Since only a few studies have examined veterinary decision-making for obesity, the reasons for the observed underreporting are not known. However, better understanding the reasons might allow strategies for change to be identified within the profession. One possible explanation for why the OW status was not recorded would be the time constraints encountered in general practice; in this respect, there might be other problems requiring more urgent attention. This is, perhaps, compounded by the fact owners rarely present their pet to the veterinarian because they are worried about excess body weight^(^^6^^)^, which itself might be related to the increased tendency for owners of OW dogs to underestimate their body condition, than owners of ideal weight dogs^(^^5^^–^^7^^)^. In addition, veterinarians might be reluctant to record the OW status if they perceive that they will encounter resistance from owners, or be concerned about offending the owner. This reluctance might arguably be greater when owners are themselves OW, and it is noteworthy that a positive association between BMI in owners and the body condition of their dog exists^(^^3^^,^^4^^)^.

A second aim was to determine what factors were associated with the likelihood of a veterinarian recording OW status, and this was explored in the case–control part of the study. The OW status was more often recorded when dogs presented with either osteoarthritis or lameness, which is logical given their known association in this species^(^^2^^,^^13^^)^. It also emphasises that veterinarians more commonly highlight the OW status where they perceive either an association with a current health concern, or that weight management might benefit mobility. In contrast, there was no association between dispensing of NSAID and recording of OW status. While NSAID usage was common, being dispensed in 27 % of all consultations, they were given for osteroarthritis and lameness in only a minority of cases (3 and 13 %, respectively). Therefore, the lack of association between NSAID use and recording the OW status might reasonably be explained by the fact that NSAID have a wide range of indications, including analgesia for non-orthopaedic problems and post-operative analgesia. Furthermore, NSAID were commonly dispensed during repeat prescription consultations and, in such circumstances, the OW status is unlikely to be recorded.

The OW status was also more commonly recorded when dogs were enrolled in a practice health scheme (a scheme whereby complimentary routine preventive health care and some additional veterinary fees are provided an upfront fee). This would be expected, since OW dogs might have joined such a scheme because they were OW. In addition, since such schemes focus on preventive health care, and would include discussions regarding maintaining a healthy weight, greater recording of the OW status would be expected. In contrast to this, the OW status was less commonly recorded in dogs that were listed on the practice system as microchipped; however, the reason for this association is unknown.

The study has a number of limitations, which should be considered. Firstly, it was retrospective in nature, and used data gathered from practice software; as a result, and some information is incomplete or missing. For instance, occasionally basic signalment data were missing, such that some consultations were excluded which might have been relevant. Secondly, the study's search terms mainly utilised the free text entered during the consultation. This free text quality most likely varied between practices and veterinarians, and might not have represented the actual extent of the discussions between veterinarian and client. Thirdly, the use of key words to identify consultations within this free text, particularly when identifying case animals was another limiting factor. Although an effort was made to consider all possible complete word, and shorthand terms for the OW status, some might have been missed. Thus, the results obtained might be an underestimate of the actual situation. That said, it is unlikely that this point alone would account for the discrepancy between the current prevalence of dogs being OW and obese, and the frequency of recording the OW status.

A fourth limitation was the method by which OW status was recorded for control dogs. Unfortunately, we were unable to use BCS to identify controls, because this parameter was rarely recorded, a finding consistent with other published work^(^^12^^)^. Such a problem is common to other studies of this nature^(^^20^^)^. Therefore, we chose to use bodyweight, stratified on breed, sex and neutered status, whereby dogs in the upper quartile were assumed to be OW. It is possible that this method incorrectly categorised some dogs as OW and vice versa.

Finally, there were limited breeds included in the present study due to excluding those without three matched controls and excluding all crossbreeds; results, therefore, cannot necessarily be applied to all breeds or to the crossbreed population. The three breeds of Labrador Retrievers, Jack Russell Terriers and Staffordshire Bull Terriers contributed proportionally the most consultations to the study. Owing to the Jack Russell Terrier not being recognised officially as a breed^(^^21^^)^, it appears overrepresented in the present study. Other than this, the breed spread is highly representative of the most commonly registered breeds^(^^22^^)^. We must also consider that, as dogs are often classified in the practice records based on the type they most represent and are not necessarily a pedigree of that breed, matching the breeds in the present study may not have overcome the actual variances in weight when calculating quartiles.

### Conclusion

We conclude that there is a marked underreporting of OW status in pet dogs, but the reasons for this are not clear. Various factors were related to the likelihood of a veterinarian recording OW status, the most notable when the consultation was for osteoarthritis or lameness. While this suggests that veterinarians are aware of the association between the OW status and orthopaedic disease in dogs, lesser importance is placed on identifying obese dogs without such complications. This presents a challenge for strategies that aim to prevent the development of obesity, or to manage the condition when it arises. Further work is needed to understand the reasons behind decision-making in veterinarians, when faced with an OW or obese dog.

## Supplementary material

The supplementary material for this article can be found at http://www.journals.cambridge.org/jns
